# Population genetics and phylogeography of *Tabanus bromius* (Diptera: Tabanidae)

**DOI:** 10.1186/s13071-021-04970-5

**Published:** 2021-09-06

**Authors:** Sumeyra Nur Sanal Demirci, Volkan Kilic, Serap Mutun, A. Yavuz Kilic

**Affiliations:** 1 Department of Biology, Graduate School of Science, Eskisehir Technical University, Eskisehir, Turkey; 2 Chemistry Group, Bioanalysis Laboratory, TUBITAK National Metrology Institute, Kocaeli, Turkey; 3 Department of Biology, Faculty of Science, Eskisehir Technical University, Eskisehir, Turkey; 4 Department of Biology, Faculty of Science and Art, Bolu Abant Izzet Baysal University, Bolu, Turkey; 5 Department of Biology, Faculty of Science, Anadolu University, Eskisehir, Turkey

**Keywords:** Horsefly, *COI*, ITS, *Tabanus bromius*, Tabanidae, Genetic structure, Phylogeographic

## Abstract

**Background:**

*Tabanus bromius* (Diptera: Tabanidae) is one of the most notable Tabanidae species of veterinary and medical importance distributed throughout the Palearctic region. In this study, we investigate the genetic diversity and the phylogeographic structure of *T. bromius* sampled from Turkey, Croatia, and Iran.

**Methods:**

For this purpose, a 686-base-pair (bp) fragment of mitochondrial DNA cytochrome oxidase I gene (*COI*) and 1339 bp of the nuclear DNA internal transcribed spacer (ITS) were sequenced from 247 individuals representing 15 populations.

**Results:**

The sequences generated 169 *COI* haplotypes and 90 ITS alleles. A higher haplotype/allele diversity (*h* = 0.9909 for the *COI* gene and A*d* = 0.8193 for the ITS region) compared to a low nucleotide diversity (*π* = 0.020605 for *COI* gene and *π* = 0.013667 for the ITS region), present for a high number of singleton and private haplotypes/alleles imply population expansion in the past. The results of phylogenetic analysis led to the uncovering of geographically significant groupings of lineages with regard to the entrance of the species into Anatolia and the location of major geographic barriers. According to current data, the species appears to have entered Turkey from Caucasia and Iran. A molecular clock applied to the *COI* data suggests that *T. bromius* diverged from the outgroup species nearly 8.83 million years ago, around the end of the Miocene era.

**Conclusions:**

The results of this study indicate remarkable genetic diversity across the studied range of the species. High haplotype/allele versus low nucleotide diversity and demographic analyses implied that the *T. bromius* populations have undergone a series of expansions and retreats in the past. Our current findings suggest that *T. bromius* split from outgroups around the Late Miocene. Subsequent diversification events during the climatic and environmental fluctuation times of the Late Pliocene and Early Pleistocene periods also significantly influenced the species, resulting in the formation of some major genetic lineages. The phylogenetic analyses indicate that *T. bromius* most likely entered Turkey from the Caucasus region and Iran.

**Graphical Abstract:**

**Figure:**
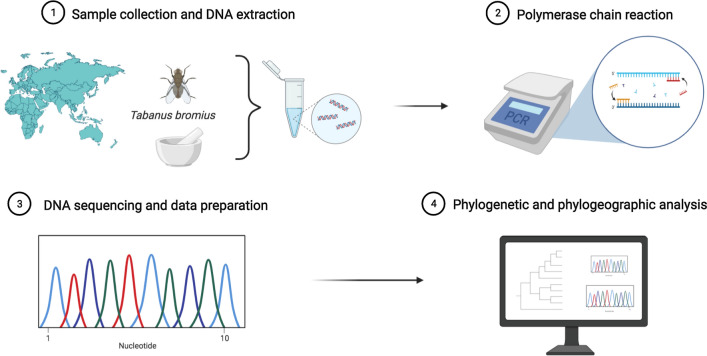

**Supplementary Information:**

The online version contains supplementary material available at 10.1186/s13071-021-04970-5.

## Background

Tabanidae comprises almost 4500 species, showing widespread distribution throughout the world, with about 660 species in the Palearctic region alone [1, 2]. Horseflies of the Tabanidae family are common blood-sucking pests of mammals and are known as important vectors of several human and animal diseases through the mechanical transmission of viruses, bacteria, protozoa, and helminths [3]. Anthrax, anaplasmosis, trypanosomiasis, and tularemia are prevalent among these diseases, and they can be fatal in certain cases if left untreated [4]. Despite their medical and economic importance as vectors and pest species, there has been limited research on Tabanidae; according to Mackerras et al. [5], it is one of the least investigated families of the order Diptera.

Investigating the geographic distribution of widespread pest and vector species is crucial for determining infectious diseases, vector control, and pest management [6]. To date, 171 horsefly species, classified under nine genera from three subfamilies, have been recorded from Turkey [7, 8]. *Tabanus bromius* is an important common pest species in the Palearctic region, including Turkey. The species was first recorded from Turkey in 1957 by Moucha and Chvála [9]. Its importance is associated with both the transmission of diseases and economic significance with its appearance in large numbers, as well as its persistence. Females suck blood from animals using their stout mouthparts and can inflict painful bites during their feeding, which can irritate grazing animals considerably, resulting in allergic responses, loss of weight, and reduction in growth rates and milk production [10, 11]. Despite its medical and economic significance, apart from a few faunistic studies [7, 8], there has been no detailed study on *T. bromius* from Turkey.

Located in the Western Palearctic region, Turkey is one of the most significant places in the world due to its high biodiversity and number of endemic species [12]. The high diversity is partly associated with its continental features changing surprisingly in short geographic distances in terms of climate and habitats [13]. In this context, both species and genetic diversity are high in Turkey, which is in line with expectations [14]. Anatolia (the Asian part of Turkey) is considered a region where much hybridization is observed due to the topography [13]. It is also the homeland of many species and has been a refuge area for those species especially affected by geological and climatic changes in the past, and it has much more biological importance than many other places in the world considering its role as a natural bridge between Europe, Asia, and Africa [15]. Moreover, the presence of areas used as a refuge by terrestrial organisms in the west and east of Anatolia during the Pleistocene period makes Turkey even more important in terms of biogeography [16, 17]. Climatic oscillations throughout the Quaternary period have particularly affected the Northern Hemisphere in different ways, and the response of species in different geographies has been dissimilar [18]. Nonetheless, studies over the last few decades suggest that Anatolia might have acted as a shelter for many species during the glaciation periods, and that these species may have spread to Europe or the Caucasus again (perhaps for the first time) during the post-glacial periods [16, 19, 20].

In recent years, there has been an increase in research involving Turkish fauna to explore the phylogenetic and phylogeographic structure of various animal groups [19–25]. Although there have been certain faunistic studies to determine Tabanidae diversity [7, 8, 26–41], there has been only one molecular-based study on horse flies in Turkey [42]. Because it has been implicated as a vector of various pathogens, and due to the lack of any molecular-based study, here we aim to investigate the population genetic structure, phylogeography, and demographic history of *T. bromius* to gain greater insight into this species. Therefore, in the present study, we use cytochrome oxidase I (*COI*) of the mitochondrial genome and the internal transcribed spacer (ITS) region of the nuclear genome as the combination to (i) reveal the genetic diversity of *T. bromius*; (ii) determine the distribution of genetic variation across the sampled area; (iii) reconstruct phylogenetic trees to reveal evolutionary relationships of haplotypes/alleles; (iv) estimate divergence of lineages; and (v) reveal possible factors that generate the contemporary phylogeographic pattern in *T. bromius.* Our results contribute to both the fauna of Tabanidae in Turkey and the vector epidemiology.

## Methods

### Sample collection and DNA extraction

**Fig. 1:**
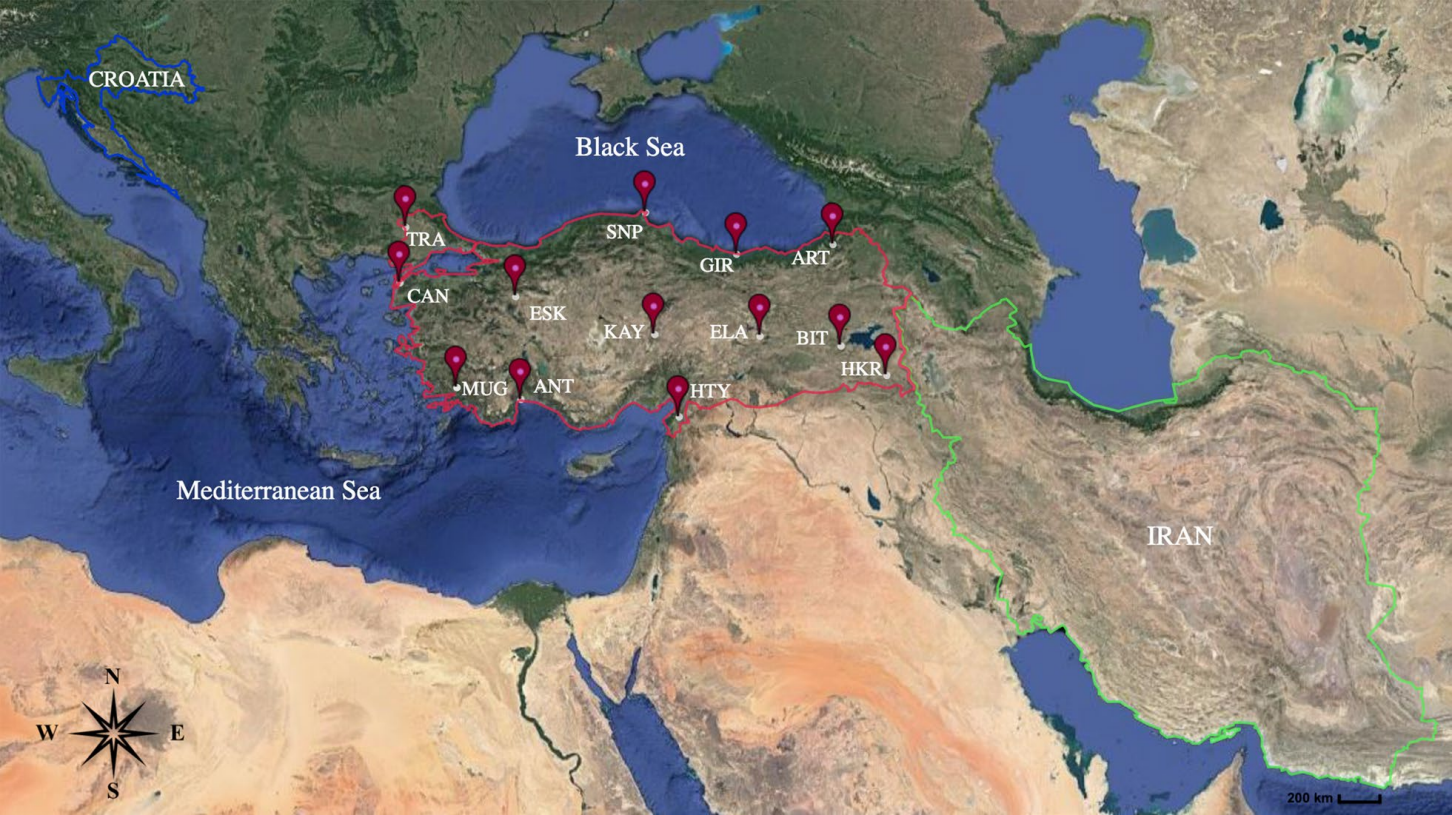
Sampling sites of *Tabanus bromius* (map by Google Maps). Population abbreviations are shown in Table 1

*Tabanus bromius* females were collected from the 13 locations (Antalya, Artvin, Bitlis, Çanakkale, Elazığ, Eskişehir, Giresun, Hakkari, Hatay, Kayseri, Muğla, Sinop and Thrace) covering most of the distribution range of the species in Turkey. Fresh specimens of *T. bromius* were caught using a malaise trap or collected manually while sucking blood from cattle. We also used museum materials registered in the inventory of the Zoology Museum of Eskişehir Technical University, Faculty of Science Department of Biology. Specimens from Iran and Croatia were also included in this study (Fig. 1). Despite all our efforts, we were not able to obtain specimens from other parts of the *T. bromius* distribution range from other countries. Therefore, in this study, a total of 247 samples (83 specimens were museum materials and 164 were fresh samples) representing 15 populations (234 samples from Turkey, 10 specimens from Croatia, and three individuals from Iran) were used to isolate the total DNA. All sampling locations, their abbreviations, and the sample size of the studied populations are given in Table 1. The old museum materials were preserved in ethyl acetate, which is known to cause problems in DNA extraction [43, 44]. Therefore, we modified [45] the total genomic DNA isolation method. After extraction, DNA samples were run at 120 V and 50 mA in 1% agarose gel, using 1× TAE (40 mM Tris pH 7.6, 20 mM acetic acid, 1 mM EDTA) buffer. The extracted DNA samples were stored at −20 °C until DNA amplification.

**Table 1:** *Tabanus bromius* populations with their abbreviations, coordinates, sample size

Population	Abbreviations	Coordinates	Sample size (no. of haplotypes/alleles)		Haplotype diversity		Nucleotide diversity	
			*COI*	ITS1-ITS2	*COI*	ITS1-ITS2	*COI*	ITS1-ITS2
Antalya	ANT	36°51'N, 31°31'E	15/10	15/9	0.9143 ± 0.0559	0.8857 ± 0.0686	0.0095 ± 0.0053	0.0021 ± 0.0013
Artvin	ART	41°21'N, 41°41'E	20/11	17/9	0.8895 ± 0.0522	0.8456 ± 0.0699	0.0066 ± 0.0038	0.0479 ± 0.0244
Bitlis	BIT	38°22'N, 41°54'E	4/4	4/2	1.0000 ± 0.1768	0.5000 ± 0.2652	0.0221 ± 0.0150	0.0008 ± 0.0008
Çanakkale	CAN	40°02'N, 26°27'E	22/19	20/3	0.9827 ± 0.0208	0.5105 ± 0.0907	0.0130 ± 0.0069	0.0008 ± 0.0006
Elazığ	ELA	38°30'N, 39°06'E	14/12	20/13	0.9780 ± 0.0345	0.8842 ± 0.0666	0.0177 ± 0.0095	0.0121 ± 0.0063
Eskişehir	ESK	39°16'N, 30°32'E	21/15	20/12	0.9619 ± 0.0260	0.8895 ± 0.0548	0.0116 ± 0.0062	0.0437 ± 0.0220
Giresun	GIR	40°31'N, 38°16'E	25/20	20/6	0.9733 ± 0.0223	0.7842 ± 0.0638	0.0070 ± 0.0039	0.0598 ± 0.0300
Hakkari	HKR	37°42'N, 48°43'E	19/10	20/9	0.8947 ± 0.0437	0.8211 ± 0.0727	0.0156 ± 0.0083	0.0124 ± 0.0064
Hatay	HTY	36°05'N, 36°02'E	8/3	18/4	0.7143 ± 0.1227	0.3137 ± 0.1376	0.0049 ± 0.0032	0.0101 ± 0.0053
Kayseri	KAY	37°05'N, 28°26'E	21/18	25/5	0.9810 ± 0.0225	0.4800 ± 0.1172	0.0141 ± 0.0075	0.0035 ± 0.0020
Muğla	MUG	37°05'N, 28°26'E	21/18	20/3	0.9810 ± 0.0225	0.1947 ± 0.1145	0.0160 ± 0.0084	0.0003 ± 0.0003
Sinop	SNP	42°01'N, 35°05'E	23/20	20/15	0.9881 ± 0.0163	0.9632 ± 0.0282	0.0233 ± 0.0120	0.0269 ± 0.0136
Thrace	TRA	41°38'N, 26°31'E	7/5	15/4	0.8571 ± 0.1371	0.4667 ± 0.1478	0.0144 ± 0.0086	0.0157 ± 0.0083
Croatia	HRV	45°08'N, 15°21'E	10/9	10/6	0.9778 ± 0.0540	0.8444 ± 0.1029	0.0132 ± 0.0075	0.0084 ± 0.0050
Iran	IRN	38°51'N, 44°37'E	3/3	3/2	1.0000 ± 0.2722	0.6667 ± 0.3143	0.0252 ± 0.0194	0.0038 ± 0.0032

The haplotype and nucleotide diversity (± standard deviation) of each population are shown in the upper and the lower part of the *COI* and ITS1-ITS2, respectively

### Polymerase chain reaction (PCR), sequencing, and data preparation

A 686 base pair (bp) of the mitochondrial *COI* gene was amplified using LCO1490 (5′-GGT CAA CAA ATC ATA AAG ATA TTG G-3′) and HCO2198 (5′-TAA ACT TCA GGG TGA CCA AAA AAT CA-3′) primer pair [46]. Amplification conditions for the *COI* gene were set as follows: 5 min at 95 °C for initial denaturation; 35 cycles of 35 s at 94 °C for denaturation; 45 s at 42 °C for annealing; 1 min at 72 °C for elongation, and 7 min at 72 °C for final extension. For amplification of the ITS (ITS1-5.8S rRNA-ITS2) region of nuclear DNA, CS249 5′-TCG TAA CAA GGT TTC CG-3′ and FL 5′-GCT GCA CTA TCA AGC AAC-3′ [47], CAS18sF1 5′-TAC ACA CCG CCC GTC GCT ACT A-3′ and CAS5p8sB1d 5′-ATG TGC GTT CRA AAT GTC GAT GTT CA-3′, CAS5p8sFt 5′-TGA ACA TCG ACA TTT YGA ACG CAT AT-3′ and CAS28sB1d 5′-TTC TTT TCC TCC GCT TAG TAA TAT GCT TAA-3′ [48] primers were used. The amplification protocol for the ITS region is shown in Additional file 1: Table S1. All PCR amplifications were carried out in a total volume of 25 µl, including 2.5 µl of 10X buffer, 2.5 µl MgCl_2_ (25 mM), 2.5 µl of each primer (2.5 mM), 2.5 µl bovine serum albumin (BSA, 10 mg/ml), 0.5 µl dNTPs (10 mM), 0.2 µl of Taq polymerase (5 U/µl) (Thermo Fisher, USA), and 1 µl of template DNA. All of the PCR products were purified using a GeneJET gel extraction kit (Thermo Fisher, USA) according to the manufacturer’s instructions. The amplicons (*COI*-ITS) were sent to a company (Macrogen, Europe) for sequencing in both directions to reduce the possibility of base-calling errors.

Chromatograms sent by the company were first visually checked, and subsequently transferred to Geneious 11.2.1 software [49] for editing, alignment, and the collapse of similar sequences into haplotypes/alleles. To ensure that the obtained *COI* sequences were genuine mitogenomes in their origin, internal stop codons and nonsense mutations were carefully checked after translating the sequences into amino acids using DnaSP 5.10.1 software [50]. All haplotypes/alleles were deposited in GenBank (MK941663-MK941831 for the *COI* haplotypes and MK936073-MK936162 for the ITS1-5.8S-ITS2 alleles). Despite all our efforts, we were not able to amplify the *COI* gene of 14 of the Turkish museum specimens, and we, therefore, included only 233 *COI* sequences and 247 ITS sequences in our data analysis. Sequences from five other tabanid species were used as outgroups: *Tabanus atratus* (GenBank: KM243515), *T. rufofrater* (GenBank: DQ631993), and *T. bifarius* [42] for the *COI* haplotypes, *T. bifarius*-1 and *T. bifarius*-2 [42] for the ITS alleles.

### Estimating genetic diversity and population structure

Molecular diversity indices, including the number of polymorphic sites (S), nucleotide (*π*) and haplotype (*h*) diversity [51], the number of substitutions, and the pairwise nucleotide differences (k) [52], were estimated using the DnaSP 5.10.1 [50] and Arlequin 3.5.2.2 programs [53]. Pairwise comparisons to determine genetic differentiation between populations were estimated using the fixation index, *F*_ST_, and their significance was tested by 10,000 permutations. The hierarchical distribution of genetic diversity was calculated as an analysis of molecular variance (AMOVA) with 20,000 permutations, with statistical significance of *P* ≤ 0.01, using the Arlequin 3.5.2.2 program [53]. The analysis was performed at three levels: among groups, among populations within groups, and within each *T. bromius* population. Population demographic analyses and any deviations from neutrality were characterized using mismatch distribution analysis as implemented in DnaSP 5.10.1 [50], the sum of squared deviations (SSD), raggedness index (*Hri*) [54], Tajima’s *D* [55], and Fu’s *F*_*S*_ [56]. Statistical significance was generated using 1000 simulations, and the significance level was set to *P* ≤ 0.05.

### Phylogenetic and phylogeographic analyses

Maximum parsimony (MP), maximum likelihood (ML), and Bayesian inference (BI) were used to infer phylogenetic relationships among *T. bromius* haplotypes/alleles using PAUP* 4.0b10 [57] and MrBayes 3.2.6 [58], respectively. An MP analysis was performed under heuristic search options using the TBR branch-swapping algorithm of random addition of taxa and was employed with 1000 bootstrap replicates [57]. The JModeltest-2 [59, 60] was used to find the best-fit model of substitution to our data sets. The GTR + I + G and GTR + G were determined as the best-fit substitution models for the mitochondrial *COI* gene and the nuclear ITS1-ITS2 region, respectively. The divergence time of the *COI* haplotypes and their confidence intervals were generated using a Bayesian Markov chain Monte Carlo (MCMC) method by applying the GTR + I + G model following the uncorrelated relaxed lognormal clock, as implemented in the BEAST version 1.5.2 [61]. The *COI* data set was calibrated by 2.3% sequence divergence per lineage per million years mutation rate [62]. Both the most ancient common ancestors (MACAs) and the most recent common ancestors (MRCAs) were obtained using BEAUTI v1.8.0 [63]. The BEAST analysis was run for 100 million generations, sampling every 1000, and the convergence to stationary and the effective sample size (ESS) of the model parameters were checked by Tracer v1.6.0. The maximum clade credibility tree was built with TreeAnnotator v1.8.4, discarding the initial 25% samples as burn-in. FigTree version 1.3.1 [64] was used to visualize the results.

Since phylogenetic trees may not always resolve evolutionary relationships among sequences due to reticulations [65], we used network analysis to analyze our data. A pairwise distance matrix between haplotypes/alleles computed by Arlequin 3.5.2.2 was transferred to HapStar version 0.5 (C) [66] separately to build a minimum spanning tree. The resulting .svg file was transferred to Inkscape 0.91 (www.inkscape.org) to visualize the networks.

## Results

### Genetic diversity of *T. bromius*

All of the sequences of the *COI* gene of 233 *T. bromius* specimens from Turkey, Iran, and Croatia generated 169 unique sequences. A total of 220 *T. bromius* samples from the Turkish localities yielded 157 haplotypes, 10 specimens from Croatia generated nine haplotypes, and three samples from Iran produced three distinct sequences. The haplotypes and their frequencies are shown in Additional file 2: Table S2. In 169 haplotypes, there were 117 polymorphic characters (17.0%), of which 94 (13.7%) were parsimony informative. The nucleotide frequencies were 38.0% (T), 29.5% (A), 16.7% (G), and 15.5% (C), respectively. The transition/transversion rate ratios were *k1* = 5.1 (purines) and *k2* = 6.0 (pyrimidines). When the *COI* sequences were translated into 228 amino acids, only 12 amino acids showed polymorphism. Pairwise differences between the haplotypes ranged between 1 bp (0.1%) and 39 bp (5.9%). Haplotype (*h*) diversity in *T. bromius* was *h* = 0.9909 ± 0.1091, and the nucleotide diversity was *π* = 0.020605 ± 0.012850. Among all of the *COI* haplotypes, seven haplotypes were shared between populations, and there were 139 singleton and 23 private haplotypes. In the Turkish populations, there were 128 singleton and 22 private haplotypes. However, in the Croatian population, there were eight singleton haplotypes, and there was only one private haplotype. Of the Iranian specimens, all three produced three unique haplotypes. No haplotypes were shared among Turkey, Croatia, and Iran.

The amplified size of the nuclear genome of *T. bromius*, including the ITS1 + 5.8S rRNA + ITS2 region, was 1339 bp in length. Since the 5.8S region (123 bp) showed no polymorphism, we eliminated it from our data set; therefore, only the ITS1 and ITS2 regions were combined and used to determine *T. bromius* alleles. Therefore, the concatenated sequence of the ITS1-ITS2 region was 1216 nucleotides in size without any length differences among 247 samples, which were collapsed into 90 unique alleles (Additional file 3: Table S3). A total of 234 Turkish *T. bromius* individuals yielded 84 alleles, while the 10 Croatian samples generated six and the three Iranian specimens produced two distinct alleles. In the ITS alleles, 538 characters (44.0%) showed polymorphism, of which 390 sites (32.0%) were parsimony informative. The average base composition was 43.0% (A), 35.0% (T), 12.4% (G) and 9.4% (C), and the transition to transversion ratio was R = 1.38. The pairwise difference between alleles ranged from 1 bp (0.1%) to 326 bp (3.3%). The average allele diversity was A*d* = 0.8193 ± 0.1417 and the nucleotide diversity was *π* = 0.013667 ± 0.007385. The numbers of singleton and private alleles were high, where 63 alleles were singleton, 23 were private alleles, and only four alleles were shared among the localities. The number of singleton alleles was 59, while the number of private alleles was 21 in the Turkish populations. In the Croatian population, four alleles were determined as singleton alleles, with one private and one shared allele being found. All of the Iranian specimens produced one private and one shared allele. There was no singleton allele in the Iranian population.

### Demographic history of *T. bromius* populations

**Fig. 2:**
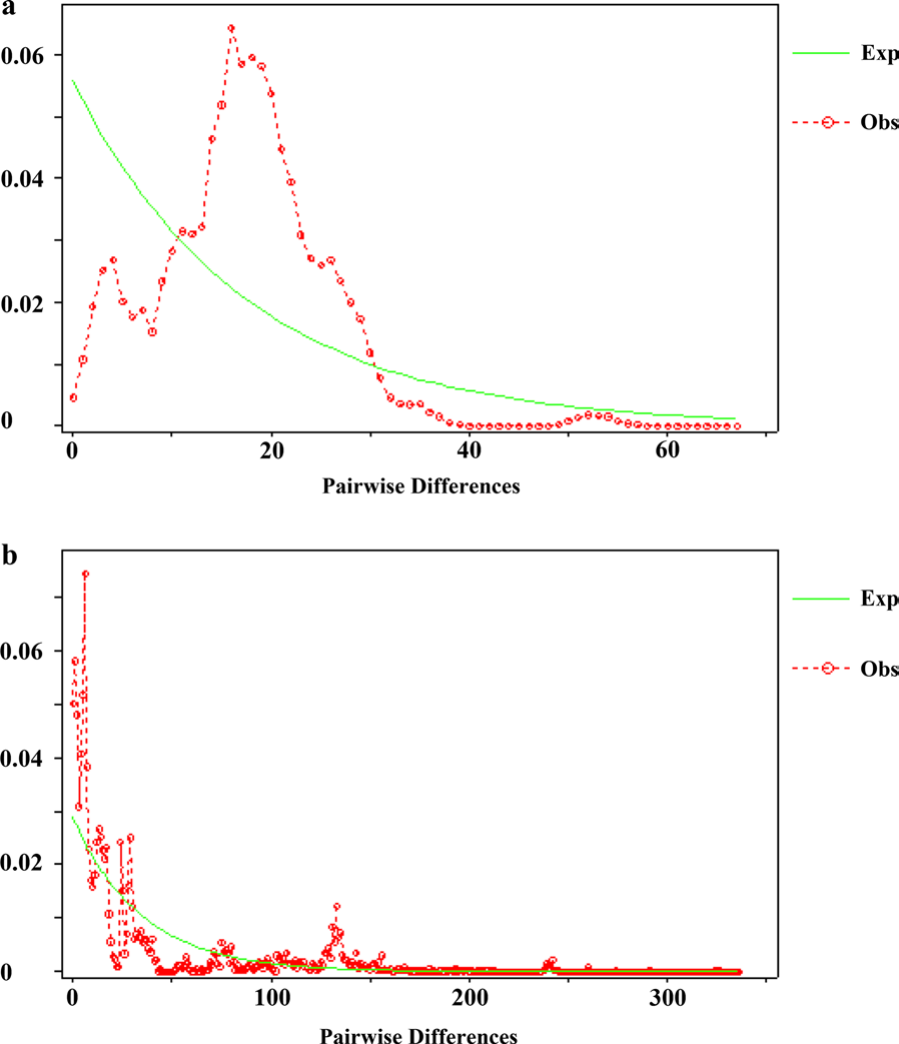
Mismatch distribution of all pairwise combinations. **a** *COI* haplotypes. **b** ITS1-ITS2 alleles. The observed distribution is represented by a red line (Obs), and the expected frequencies by a green line (Exp)

Deviations from neutrality and population size changes were investigated through several analyses. Analyses of all of the *COI* haplotypes produced significantly negative Fu’s *F*_*S*_ values (*F*_*S*_ =  −23.7386, *P* ≤ 0.05), implying population expansion. However, Tajima’s *D* was not significant (Tajima’s *D* =  −0.4694, *P* ≥ 0.05). Harpending’s raggedness index *Hri* = 0.0011 and SSD = 0.0038 were low. Individually analyzed populations showed that a number of the Turkish populations, such as Çanakkale (*F*_*S*_ = −7.8601, *P* ≤ 0.05), Giresun (*F*_*S*_ =   −12.964, *P* ≤ 0.05), Kayseri (*F*_*S*_ =  −6.3779, *P* ≤ 0.05), Muğla (*F*_*S*_ =  −5.5835, *P* ≤ 0.05) and Sinop (*F*_*S*_ =  −4.7583, *P* ≤ 0.05) expanded in the past, while all other remaining populations produced no significant values (Table 2). Similarly, Tajima’s *D* and Fu’s *F*_*S*_ values of individually analyzed Croatian and Iranian localities were not statistically significant. On the other hand, a mismatch distribution analysis of all of the *COI* haplotypes produced a bimodal profile (Fig. 2a). Individually analyzed Turkish populations showed that the Eskişehir and Iran populations generated bimodal curves; however, the remaining populations produced multimodal curves (Additional file 4: Figure S1).

**Table 2:** Fu's *F*_*S*_, Tajima’s *D*, *Hri* and SSD values calculated for each population

*COI* gene					ITS1-ITS2 region			
Population^a^	Fu’s *F*_*S*_	Tajima’s *D*	*Hri*	SSD	Fu’s *F*_*S*_	Tajima’s *D*	*Hri*	SSD
ANT	−1.2337	−0.4553	0.1223	0.0682	−3.4149*	1.3776	0.1326	0.0403
ART	−2.0402	−0.9925	0.0140	0.0050	10.8843	1.6225	0.0766	0.0690
BIT	0.8093	0.3078	0.2222	0.0849	1.0981	−0.7099	0.7500	0.1892
CAN	−7.8601*	−0.4538	0.0222	0.0179	1.3211	−1.5513*	0.1818	0.0245
ELA	−2.0062	−0.6920	0.0282	0.0331	0.3472	−2.1145*	0.0185	0.0164
ESK	−3.3836	−0.8866	0.0257	0.0382	7.3342	−1.6322*	0.0482	0.0353
GIR	−12.964*	−0.3048	0.0347	0.0095	26.2325	0.8564	0.2793*	0.1272
HKR	1.4614	1.3288	0.0334	0.0335	4.3821	1.8011	0.1053*	0.0704
HTY	2.8952	0.5378	0.4489	0.1039	11.3181	−2.5732*	0.3917	0.0337
KAY	−6.3779*	0.0001	0.0476	0.0402	4.0410	0.3239	0.2114	0.2810
MUG	−5.5835*	−0.1305	0.0056	0.0044	−0.6255	−1.8678*	0.5308	0.0115
SNP	−4.7583*	−0.2998	0.0159	0.0138	1.2514	1.1930	0.0183	0.0224
TRA	1.6472	−0.3784	0.1700	0.0699	13.180	−0.9582	0.2960	0.2844
HRV	−1.8671	0.7842	0.0864	0.0355	0.9801	0.3023	0.0873	0.0371
IRN	1.7162	−0.0000	0.4444	0.2580	2.8841	0.0000	1.0000	0.3364

*P-value ≤ 0.05

^a^Population abbreviations are shown in Table 1

For the ITS1-ITS2 data sets for all of the populations, Tajima’s *D* value was significantly negative (Tajima’s *D* = −1.9563, *P* ≤ 0.05), implying deviations from neutrality, while Fu’s *F*_*S*_ (*F*_*S*_ = −5.5854, *P* ≥ 0.05) value was non-significant. *Hri* (0.0045) and SSD (0.0040) values calculated for all of the alleles were low. When each population was analyzed separately, Fu’s *F*_*S*_ values were non-significant for all of the populations, except for Antalya. The Antalya population also suggested population growth by a significantly positive value (Fu’s *F*_*S*_ = −3.4149, *P* ≤ 0.05). The Çanakkale (Tajima’s *D* = −1.5513, *P* ≤ 0.05), Elazığ (Tajima’s *D* = −2.1145, *P* ≤ 0.05), Eskişehir (Tajima’s *D* = −1.6322, *P* ≤ 0.05), Hatay (Tajima’s *D* = −2.5732, *P* ≤ 0.05), and Muğla (Tajima’s *D* = −1.8678, *P* ≤ 0.05) populations generated significantly negative values (Table 2). Fu’s *F*_*S*_ and Tajima’s *D* values calculated for the Croatian and Iranian populations were not statistically significant. Other than this, all of the ITS1-ITS2 datasets produced a multimodal curve (Fig. 2b). While the Antalya, Bitlis, Çanakkale, Muğla, and Iran populations showed bimodal curves, the remaining populations generated multimodal curves, and all these populations suggested either stable or diminished populations (Additional file 5: Figure S2).

### Phylogenetic and phylogeographic analyses

**Fig. 3:**
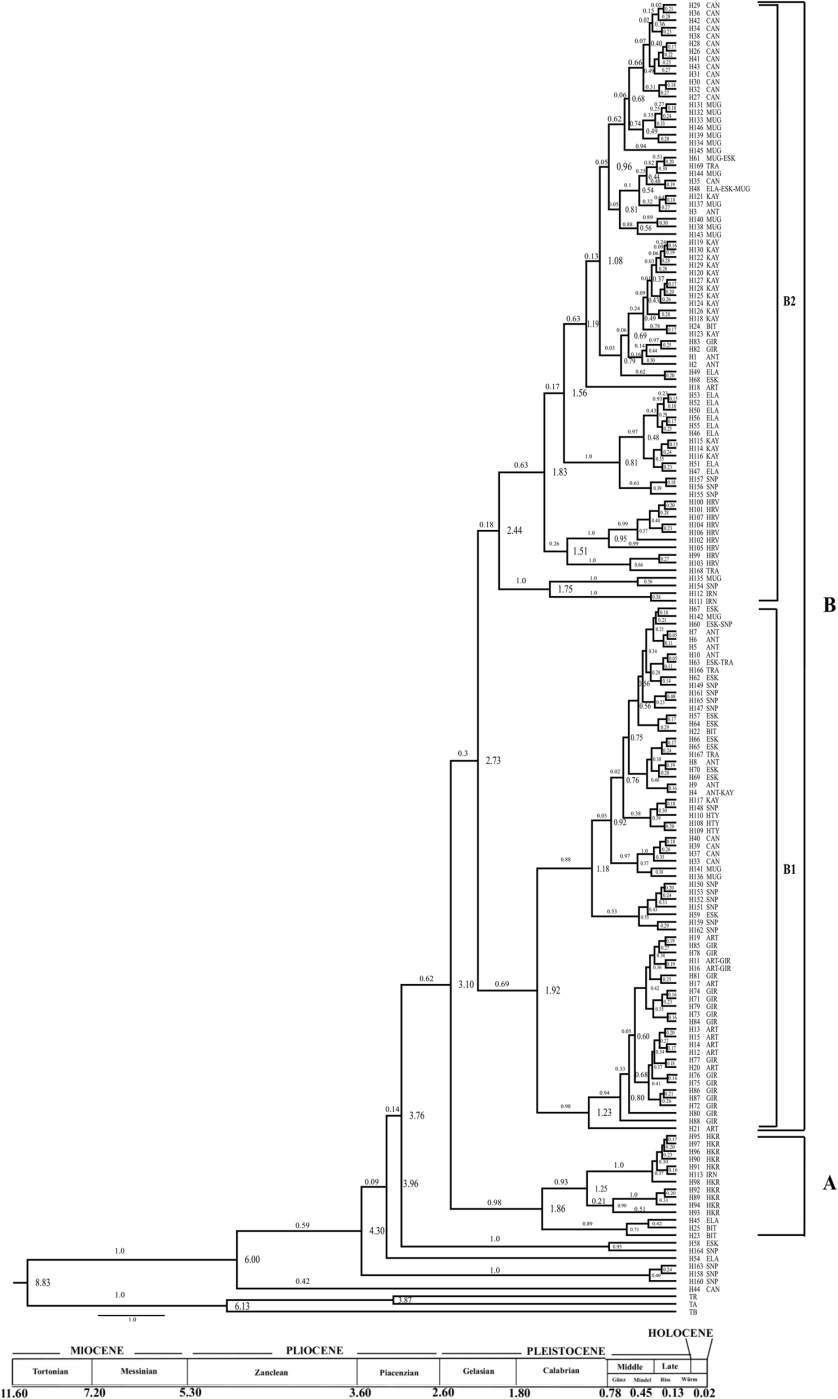
The BEAST tree of *COI* gene haplotypes. Node ages (tMRCA) are shown in the interior part of each related node and posterior probability values are also on the branches. Population abbreviations are shown in Table 1

All three analyses conducted for both data sets produced similar tree topologies with different bootstrap/posterior probability values. Therefore, only Bayesian consensus trees were provided here for both the *COI* haplotypes and ITS1-ITS2 alleles (Figs. 3, 5). The resulting BEAST tree is also compatible with other trees. For the *COI* data set, all of the trees produced two main clades; the first clade (Clade A) comprised an Iranian haplotype (H113) and Turkish haplotypes from the eastern part of Turkey (Hakkari, Elazığ, and Bitlis). The second main clade (Clade B) splits into two subclades (B1 + B2); the subclade B1 with a northern basal haplotype (H21) sampled from the Artvin population, and most of the remaining haplotypes were from the Giresun and Artvin populations. Moreover, the subclade BI is composed of haplotypes representing eastern, central, northern, and western Turkey. The subclade B2 has a basal haplotype (H111) sampled from the Iranian population, with the remaining haplotypes from Croatia, and it is mainly dominated by the western and central Turkish haplotypes.

Application of a molecular clock showed that the ingroup haplotypes of *T. bromius* diverged from the outgroup haplotypes around 8.83 million years ago (MYA). The separation of the most basal haplotype (H44) from the main clade was around 6.00 MYA (Clade A). A small clade formed by Sinop haplotypes (H160, H158, and H163) located in northern Turkey diverged from the main clade nearly 4.30 MYA. The Iranian and eastern Turkish haplotypes (Hakkari, Elazığ, Bitlis) appear to have diverged from each other around 3.10 MYA. The separation of the B clade into the subclades B1 + B2 was about 2.73 MYA. The subclade B1 has a northern basal haplotype (H21) from the Artvin population. The remaining haplotypes are from certain Black Sea localities, in addition to the central, northern, and western Turkish populations. One of the Iranian haplotypes (H113), along with the Hakkari haplotypes (from eastern Turkey) formed a small clade. This haplogroup diverged from other eastern (Elazığ and Bitlis) haplotypes around 1.86 MYA. The remaining haplotypes from Iran separated from the Turkish and Croatian haplotypes around 2.44 MYA. In subclade B2, the Iranian (H111, H112) and Turkish haplotypes (H154 from Sinop, and H135 from Muğla) split from other Turkish haplotypes about 1.75 MYA. All of the Croatian haplotypes were grouped with a Turkish haplotype (H168) from the Thracian part of Turkey (the European part of Turkey), which was placed at the basal part of subclade B2. The subclade separation around 1.82 MYA implies early Pleistocene diversification. Further splitting events continued throughout the Pleistocene period generating intermediate to shallow splits creating small *T. bromius* haplogroups.

**Fig. 4:**
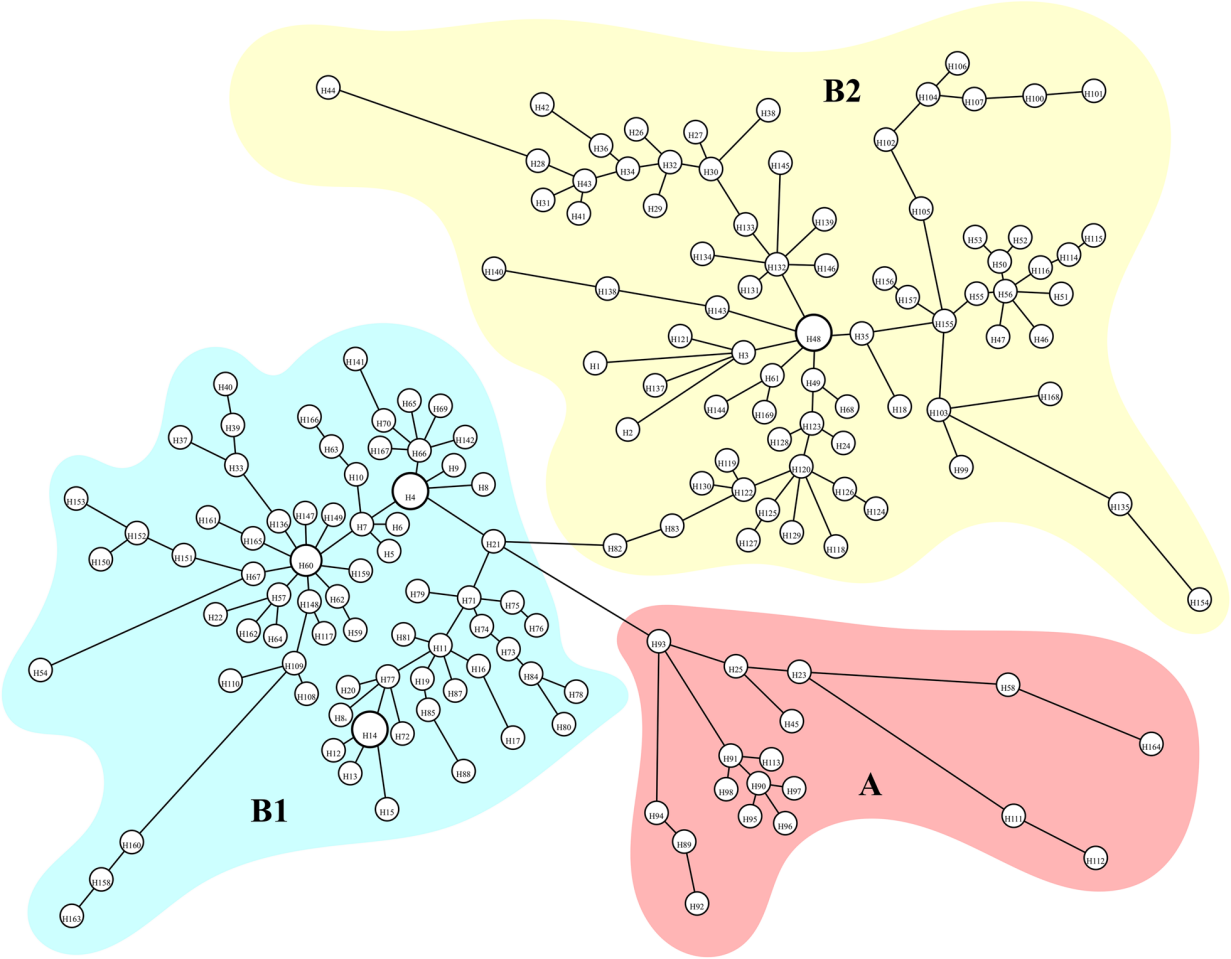
The results of the minimum spanning network analysis of the *COI* haplotypes of *T. bromius* using HapStar 0.7. Size of the circles is proportional to the frequency of the haplotype. The branch lengths are proportional to the number of hypothetical ancestors. Haplotypes are shown in Additional file 2: Table S2

The minimum spanning network analysis supports the general pattern revealed by the phylogenetic trees of the *COI* gene (Fig. 4). Three main haplogroups are apparent in the network. The first shown by A in the network is coherent with Clade A in the phylogenetic trees. The haplogroup in this cluster is composed of both Iranian (H113) and predominantly eastern Anatolian haplotypes. The other haplotypes that formed subclade B1 and B2 in the BEAST tree are also obvious as B1 and B2 in the network. The B haplogroup is dominated by haplotypes from the central, northern, and western Anatolian populations and a small star phylogeny has H21 as the central haplotype detected from Artvin, the locality that is located in the north eastern part of Turkey. The B2 haplogroup comprises haplotypes, mostly from western and central Turkey. H48, a shared haplotype among Elazığ, Eskişehir, and Muğla with the highest frequency, is connected to other haplotypes found mainly in the western Anatolian populations.

**Fig. 5:**
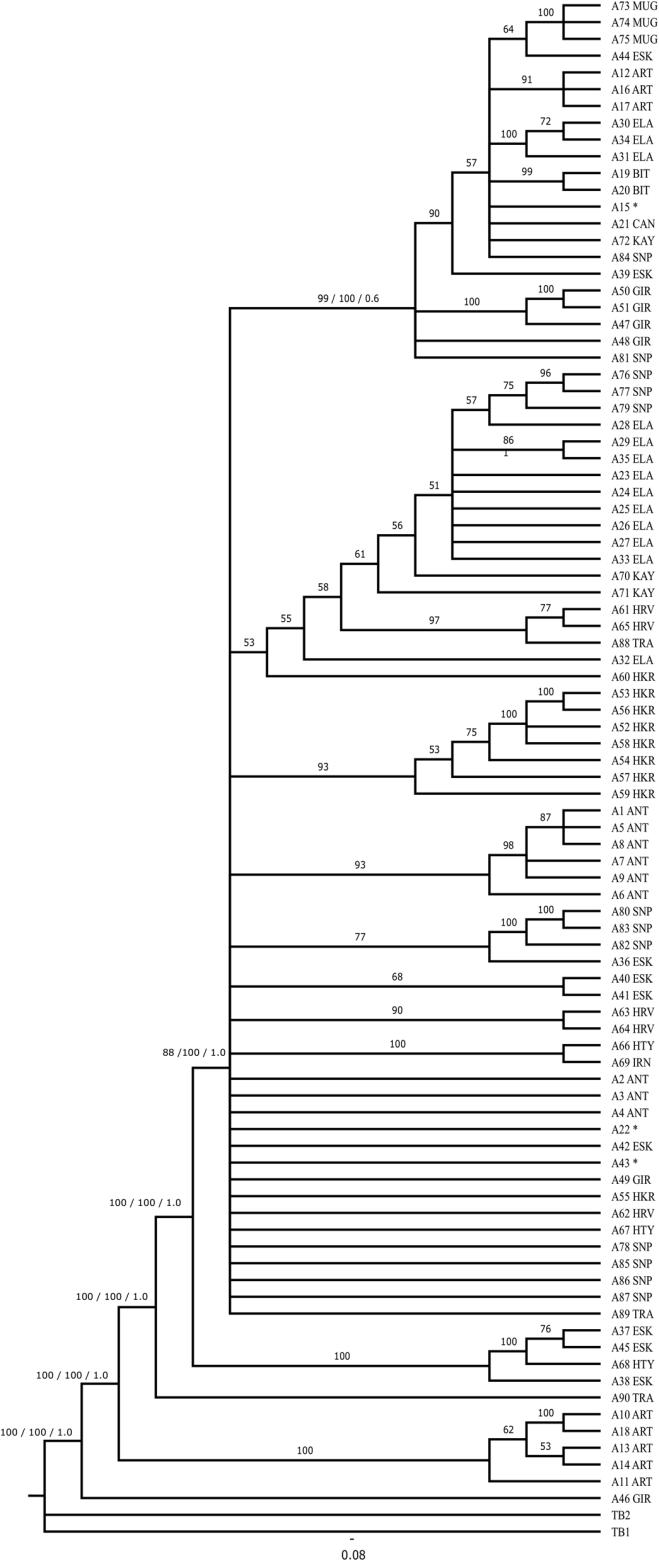
Bayesian consensus tree of ITS1-ITS2 alleles with branch posterior probabilities shown in the interior part of each related branch. Values on the branches indicate bootstrap values for the MP/ML and the posterior probability values, respectively. Population abbreviations are shown in Table 1

All three phylogenetic analyses of the concatenated ITS1-ITS2 region of *T. bromius* generate similar trees with different bootstrap/posterior probability values; therefore, only a single tree is provided here (Fig. 5). Analysis of the ITS1-ITS2 region yields predominantly polytomous structure with relatively low resolution. The A46 allele (from Giresun), at the most basal of the tree, and the A11 allele (from Artvin) form a small clade. There is an A90 allele (from the Thrace region of Turkey) at the most basal of the clade showing polytomy. The clade is divided into two sister subclades; the first subclade is composed of Eskişehir and one Hatay (A68) allele, while the other main clade structure consists of many polytomic alleles or allele groups. Furthermore, in the main clade, the Croatian (A63–A64) and the Turkish alleles from Hakkari, Antalya, Sinop, and Eskişehir (A40–A41) are grouped together. The Iranian and Hatay alleles form a monophyletic group, and all of the remaining alleles are from the eastern, central, northern, and western parts of Turkey.

**Fig. 6:**
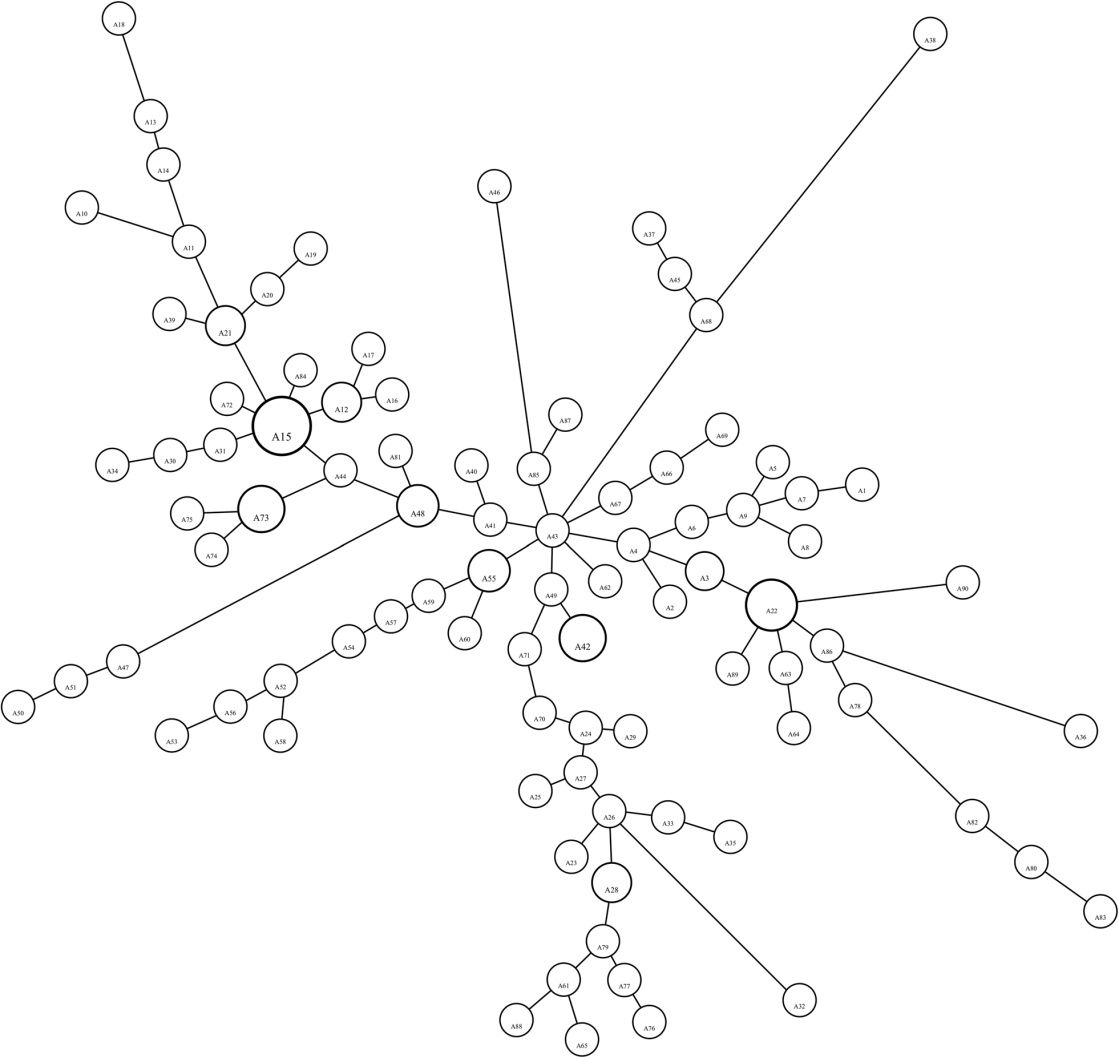
The results of the minimum spanning network analysis of the combined ITS1-ITS2 region of *T. bromius* using HapStar 0.7. Size of the circles is proportional to the frequency of the allele. The branch lengths are proportional to the number of hypothetical ancestors. Allele are shown in Additional file 3: Table S3

A minimum spanning network of the ITS region is given in Fig. 6. A15 (*n* = 40 individuals), being the most frequent and most common allele shared among the Artvin, Çanakkale, Eskişehir, Kayseri, and Sinop populations of *T. bromius*, produces a star-shaped structure. A22 is also a shared allele among Turkey (Çanakkale, Eskişehir, Sinop, and Thrace), Croatia, and Iran. It is remarkable that the A43 allele (*n* = 1 from Eskişehir) is connected to eight alleles, including the southern, northern, eastern, and western Turkish populations, and Croatia.

### Partitioning of genetic variation and population differentiation

The pairwise *F*_ST_ values show significant genetic differentiation for the *COI* data set (Additional file 6: Table S4). The highest *F*_ST_ value is between Iran and Giresun (from northern Turkey *F*_ST_ = 0.759, *P* ≤ 0.05). Iran also produces the next highest statistically significant *F*_ST_ value with another northern Turkish population (from Artvin) (*F*_ST_ = 0.751, *P* ≤ 0.05). In addition, the Iranian population is genetically different from other Turkish populations (Antalya, Çanakkale, Eskişehir, Kayseri, Hatay). On the other hand, Iran-Hakkari and Iran-Bitlis populations, which are geographically relatively close to each other, produce low *F*_ST_ values (*F*_ST_ = 0.343 and *F*_ST_  = 0.312, *P* ≤ 0.05, respectively). For the Croatian population, the highest *F*_ST_ is observed in comparisons between Croatia and Artvin (*F*_ST_ = 0.68, *P* ≤ 0.05), and Giresun (*F*_ST_ = 0.674, *P* ≤ 0.05), respectively. Pairwise comparisons also show that the highest differentiation between the Turkish populations is between Çanakkale and Artvin (*F*_ST_ = 0.684, *P* ≤ 0.05), and between Çanakkale and Giresun (*F*_ST_ = 0.670, *P* ≤ 0.05), respectively.

Genetic differentiation between populations, using the ITS data sets, was also employed through pairwise *F*_ST_ analysis (Additional file 7: Table S5). The highest *F*_ST_ value was found in comparisons between Muğla and Bitlis (*F*_ST_ = 0.983, *P* ≤ 0.05), Iran (*F*_ST_ = 0.974, *P* ≤ 0.05), Çanakkale (*F*_ST_ = 0.970, *P* ≤ 0.05), Antalya (*F*_ST_  = 0.955, *P* ≤ 0.05), Croatia (*F*_ST_ = 0.925, *P* ≤ 0.05) and Kayseri (*F*_ST_  = 0.902, *P* ≤ 0.05). For the Croatian population, the highest genetic differentiation was observed between Croatia and Çanakkale (*F*_ST_ = 0.672, *P* ≤ 0.05), and Bitlis (*F*_ST_ = 0.626, *P* ≤ 0.05), respectively. For the Iranian population, the highest *F*_ST_ value was between Iran and Çanakkale (*F*_ST_ = 0.857, *P* ≤ 0.05). The lowest genetic differentiation (*F*_ST_ = 0.267, *P* ≤ 0.05) was between the Hakkari and Iranian populations, which are geographically in proximity compared to other populations.

An analysis of the hierarchical distribution of genetic diversity shows that the highest genetic variation is at the “within population” level (46.0%, *P* ≤ 0.01), followed by “among populations“ (38.3%, *P* ≤ 0.01) and “among groups“ (15.7%, *P* ≤ 0.01) for the *COI* data set when all populations are grouped under three clusters (Group1: HRV Group2: IRN, Group3: the Turkish populations of ANT, ART, BIT, CAN, ELA, ESK, GIR, HKR, HTY, KAY, MUG, SNP, TRA). The genetic differentiation between the groups is *F*_ST_ = 0.54, *P* ≤ 0.001. On the other hand, the ITS data set shows that 65.2% of the genetic variation is within populations and 34.7% is among populations when each locality is considered as a separate group. The genetic differentiation between the groups is *F*_ST_ = 0.34, *P* ≤ 0.001 (Table 3).

**Table 3:** AMOVA analysis of *Tabanus bromius*. Analysis of the *COI* data includes three groups: Group 1 (HRV), Group 2 (IRN) and Group 3 (ANT, ART, BIT, CAN, ELA, ESK, GIR, HKR, HTY, KAY, MUG, SNP, TRA). In the analysis of the ITS1-ITS2 data, each population was treated as a distinct group. Population abbreviations are shown in Table 1

Source variation	Degrees of freedom	Sum of squares	Variance components	Percentage of variation
*COI*				
Among groups	2	100.547	1.57561	15.7
Among populations	12	824.718	3.83625	38.3
Within populations	218	1003.451	4.60299	46.0
Total	232	1928.717	10.15942	
ITS1-ITS2				
Among populations	14	1529.530	6.00779	34.8
Within populations	232	2615.114	11.27204	65.2
Total	246	4144.644	17.27983	

## Discussion

### Genetic diversity and population demography of *T. bromius*

Genetic diversity estimates of the Turkish *T. bromius* populations for the *COI* region are conspicuously high. Among the Turkish populations, the Sinop population, located in the northern part of Turkey, shows the highest diversity. Similarly, the ITS1-ITS2 region also yields a considerable number of alleles and reveals a high level of genetic diversity. When only the Turkish populations are considered, two northern Anatolian populations, Sinop and Giresun, show the highest diversity estimates. Moreover, the Sinop population shows the highest genetic diversity for both the *COI* gene and the combined ITS1-ITS2 region. Therefore, it seems that Sinop is an important location for the *T. bromius* genetic variation. Similar results have been observed in another *Tabanus* species studied from Turkey [42]. In fact, the presence of high genetic diversity has been reported from other animal groups, including insects from Turkey studied [21, 67–70], and explained by its topographic structure, the presence of microhabitats, and climatic characteristics [71]. Despite the low number of samples included from Iran and Croatia, both localities also display high diversity. However, more specimens covering a larger sampling area across the distribution range of *T. bromius* are necessary.

Among 169 *T. bromius* haplotypes, we detected 139 as singleton and 23 private haplotypes. There are a number of sequences that were found either only in the Iranian or the Croatian populations. Although the number of samples is not equal among Iran, Croatia, and Turkey, there were 128 singleton and 22 private haplotypes in the Turkish populations. Despite the low number of specimens included from Iran and Croatia, it may be correct to suggest that Turkey has the conspicuous level of variation across the sampled area, and that the species has been in Turkey for a prolonged time period, so that it possessed the high number of variants. The presence of both high singleton and high private haplotype numbers imply that these haplotypes have been derived relatively recently. In addition, the high rate of derived haplotypes may be associated with the presence of young sequences in the data set of expanding populations [72]. In *T. bromius* the nucleotide diversity is low compared to the haplotype diversity for both *COI* and ITS data for the Turkish populations. It is known that the high haplotype, but low nucleotide diversity, and the private-singleton haplotypes suggest that the population has shown fluctuations in the past, and that it generally points to population growth in the more recent past following population bottlenecks [18].

The frequency and geographic distribution of *T. bromius* haplotypes/alleles imply the possible effects of certain biogeographic barriers in Turkey. For instance, the allele with the highest frequency and the most common allele (the A15 allele shared among Artvin, Çanakkale, Eskişehir, Kayseri, and Sinop in Turkey) is distributed among the Black Sea region, western, and central Anatolia. The presence of the A15 allele only in the western part of a major mountain range known as the Anatolian Diagonal dividing Anatolia into East and West [73] suggests that *T. bromius* might have also been affected by this barrier. Moreover, the absence of shared haplotypes/alleles between the eastern and western sides of the mountain line supports the importance of the Anatolian Diagonal on *T. bromius*. In fact, it has been shown that the Anatolian Diagonal plays a key role in the distribution of species and genetic lineages, not only in plants [74, 75], but also in animals [23, 25, 68, 76–78]. A geographic structuring across the distribution range of *T. bromius* in Turkey is further supported by the pairwise *F*_ST_ analysis predicting the levels of genetic differentiation of populations and reveals significant differences from each other in many populations. Iran is one of the entrance doors into Turkey of *T. bromius* and the population representing this region is quite different from the Black Sea populations (Giresun and Artvin), while they differ slightly from the eastern populations (Hakkari and Bitlis). Another entrance gate, the Black Sea region (Artvin), has an extremely high differentiation rate with its population of Çanakkale and Croatia (representing the European region) proposing the presence of geographic separation of lineages after entering into Turkey [22, 68, 71].

The effects of historical factors can be traced by employing several population demographic analyses [18]. For *T. bromius*, the mismatch analysis of the *COI* data, where all of the populations included in the analysis generated a bimodal profile with *Hri* = 0.0011 and SSD = 0.0038, but were not statistically significant, indicates population expansion [79]. Fu’s *F*_*S*_ (−23.7386) is significantly negative, indicating an excess of rare haplotypes over what would be expected under neutrality in an expanding population is also implied by the mismatch distribution. Negative and non-significant Tajima’s *D* imply that populations of *T. bromius* did not deviate from neutrality. In our results, although Tajima’s *D* and Fu’s *F*_*S*_ value seem to conflict, the reason is different statistical power, because Fu’s *F*_*S*_ is more powerful and more sensitive than Tajima’s *D* to population size changes [80]. On the other hand, the combined ITS1-ITS2 region generated a multimodal profile with *Hri* = 0.0045 and SSD = 0.0040, but it was non-significant, indicating population expansion. For the ITS1-ITS2 region, it can be seen that the species has a stable population distribution. This is also supported by the negative significant Tajima’s *D* = −1.9563 value implying population expansion. Overall, the mismatch distribution analysis and other population demographic analyses, in addition to the combination of high haplotype and low nucleotide diversity of *T. bromius* populations for both the *COI* gene and the combined ITS1-ITS2 regions, suggest rapid demographic expansion from a small population in the past [81].

### Estimation of divergence times of *T. bromius* lineages

Horseflies are a cosmopolitan group spread across all continents and are reported to be of Pangea origin. It is thought that they emerged in the late Triassic period just before the drifting of the Laurasia and Gondwana continents [82]. Based on morphological and molecular evidence, the oldest species of a true tabanid is thought to have derived during the late Jura [83], and the emergence of the tabanid species coincided with the early Cretaceous period [84]. The Tabanidae species is rich in the equatorial belt, both in terms of species diversity and population density, but it gradually decreases in number towards the poles [10] because the adult activity period of the species increases depending on climate temperature. The majority of the terrestrial fauna of the Palearctic region including Turkey is thought to have originated from Laurasian fauna [13]. Based on the distribution of the Tabanidae species in the Palearctic region, the Turkish Tabanidae species is reportedly composed of Mediterranean, European, Asian, Eurasian, Ethiopian and endemic species [85–90]. This view is based only on the geographic distribution of the *T. bromius* species in the Palearctic region, and is not focused on any assessment of phylogeography. Our study species, *T. bromius*, shows a cosmopolitan distribution in the Palearctic region. The species has been recorded from all European countries [91] (except Ireland), North Africa (Algeria and Morocco), the Near East and a number of Middle Eastern countries, Central Asian countries (Kazakhstan, Afghanistan) up to the Northern Urals [10], India [92] and Turkey [7, 8, 91, 93]. When the distribution of both the *T. bromius* and other *Tabanus* species [1, 10] in the Palearctic region is taken into consideration, it has been estimated that the origin of the group may have been Central or West Asia, and that they might have entered Europe from north of the Black Sea (the Caucasus region), and from Turkey in the south.

Our current findings indicate that *T. bromius* diverged from outgroups approximately 8.83 MYA around the Tortonian period of the Late Miocene. During the Miocene, more than a quarter of the world was covered with pasture, which may have given the tabanids an advantage in terms of their feeding and egg laying. It is estimated that while this increased the number of warm-blooded animals feeding on this type of pasture, it also increased the density of female tabanids feeding on the blood of these animals. The Miocene was a period of intense climatic and environmental change; therefore, it is possible that the great fluctuations that occurred during the Late Miocene period may have resulted in the separation of *T. bromius* from the outgroup species. Indeed, concurrent findings have also been suggested in a number of other studies for the separation of species throughout the Miocene period [2]. It seems that after the splitting of the *T. bromius* lineage from the ancestral stock species, it has undergone certain major diversification events. Our analysis implies that the estimated age of the most basal haplotype of *T. bromius* (H44 from Çanakkale) is around 6.00 MYA, coinciding with the end of the Miocene period. In the ingroup, an eastern haplotype from Elazığ (H54) split from its sister clade almost 4.30 MYA around the Early Pliocene period. Similarly, the estimated divergence time of the two main clades (Clade A and Clade B) was approximately 3.10 MYA, which indicates the Late Pliocene period, and ongoing diversification events have continued from the beginning of the Pleistocene period until the present day. This coincides with the effects of glacial and interglacial changes in the Quaternary (in Pleistocene) [17, 94, 95]. Quaternary climatic oscillations caused the expansion or contraction of species that played an important role in shaping the genetic models of populations [96]. Such diminishing/expansion and the isolation following multiple glacial refugia can maintain high intra-genetic diversity [97]. These models can be well illustrated by a phylogeographic analysis with hereditary data in and among the population [98]. It appears that the Quaternary climate oscillations were effective in shaping the populations and lineages of the *T. bromius* species.

Our phylogenetic analyses of *T. bromius* suggest that the species most likely entered Anatolia through both the Caucasus region and Iran. The lineage that entered through Iran has shaped the eastern populations (Elazığ, Bitlis, and Hakkari) up to the Anatolian Diagonal, and the lineage that entered through the Caucasus spread westward through the Black Sea region and crossed the Anatolian Diagonal. Thereafter, Central Anatolia (Kayseri, Eskişehir), south of Turkey (Hatay and Antalya), and Western Anatolian populations (Muğla, Çanakkale and Thrace) were relatively separated in the western part of Anatolia. Furthermore, the placement of the haplogroup, consisting of the Thracian and Croatian populations in the same clade, suggests that the species provided a genetic source to the Croatian population. This suggests that the Central European *T. bromius* populations may have originated from Thracian populations or that a portion of the *T. bromius* European populations originated from the lineage from Turkey. The Black Sea region populations (Artvin and Giresun) are in subclade B1; it may be correct to assume that the collected samples are related to the distribution in a narrow area north of the North Anatolian mountain range. This is also supported by the co-grouping of Black Sea populations in one of the three-star phylogenies in the haplotype network. The Sinop population haplotypes present an interesting pattern due to phylogenetic trees, suggesting that the Sinop population is interacting with other populations. Similar results have been observed for *T. bifarius* [42]. Antalya and Hatay haplotypes formed subclade B1 together with the Central Anatolian haplotypes. In particular, all Hatay haplotypes are grouped together with Kayseri (H117) and Sinop (H148) haplotypes and, as a consequence, it has been proposed that the Hatay population might have originated from Turkey. The absence of the species in countries such as Lebanon and Jordan, close to the Hatay border, and the fact that the lineage relationship with the Hakkari population is not seen in the phylogenetic trees, may support this idea. The ITS1-ITS2 analysis produced similar phylogenetic tree topologies. There is also an allele structure with geographically significant groupings among the polytomic groups and Artvin and Giresun alleles that are found at the basal part of all tree topologies. In addition, due to the presence of the *T. bromius* species in countries such as Russia and Ukraine, it may be assumed that a lineage in the north of the Black Sea (from the Caucasian), which was a freshwater lake at that time, reached Europe. Moreover, these results agree with our results that the Turkish *T. bromius* populations originated in the Caucasus region (Artvin) and Iran.

## Conclusions

In conclusion, the *T. bromius* populations show quite remarkable genetic diversity. Demographic analyses and high haplotype/allele versus low nucleotide diversity imply that the *T. bromius* populations might have undergone a series of expansions in the past. The *T. bromius* haplotypes split from outgroup species around the Miocene period, and current results clearly indicate that the structuring of *T. bromius* genetic lineages was significantly influenced by climatic and environmental fluctuations during the Late Pliocene and Early Pleistocene periods. The phylogenetic analysis findings show that the species appears to have entered Turkey from Caucasia and Iran, and that the presence of geographic barriers, such as the Anatolian Diagonal, separates geographically important lineage groupings. Larger sampling across the western Palearctic region and a more detailed study are necessary to draw a general conclusion regarding the phylogeographic structure of *T. bromius*.

## Supplementary Information

**Additional file 1**:** Table S1**. PCR cycles for the ITS (ITS1-5.8S rRNA-ITS2) region.

**Additional file 2**:** Table S2**. *COI* haplotypes and their frequencies in populations.

**Additional file 3**:** Table S3**. ITS1-ITS2 alleles and their frequencies in populations.

**Additional file 4**:** Figure S1**. Mismatch distribution profile of each population obtained using the *COI* haplotypes. The observed distribution is represented by a red line (Obs), and the expected by a green line (Exp).

**Additional file 5**:** Figure S2**. Mismatch distribution profile of each population obtained using the combined ITS1-ITS2 alleles. The observed distribution is represented by a red line (Obs), and the expected by a green line (Exp).

**Additional file 6**:** Table S4**. COI gene FST analysis results showing pairwise genetic variations between populations. *: *P* ≤ 0.01, -: not significant.

**Additional file 7**:** Table S5**. ITS1-ITS2 region FST analysis results showing pairwise genetic variations between populations. *: *P* ≤ 0.01, -: not significant.

## Data Availability

Data supporting the conclusions of this article are included within the article. Sequences used in this study are deposited in GenBank (MK941663-MK941831 for the *COI* haplotypes and MK936073-MK936162 for the ITS1-5.8S-ITS2 alleles).

## Abbreviations

*COI*: Cytochrome oxidase I gene
ITS: Internal transcribed spacer
EDTA: Ethylenediaminetetraacetic acid
AMOVA: Analysis of molecular variance
SSD: Sum of squared deviations
*Hri*: Raggedness index
MP: Maximum parsimony
ML: Maximum likelihood
BI: Bayesian inference
MCMC: Markov chain Monte Carlo
MACAs: Most ancient common ancestors
MRCAs: Most recent common ancestors
ESS: Effective sample size
MYA: Million years ago
