# Supplementary material for: Population genetics and phylogeography of Tabanus bromius (Diptera: Tabanidae)
Source: Parasit Vectors. 2021 Sep 6;14:453. doi: 10.1186/s13071-021-04970-5 (PMC8420036; doi:10.1186/s13071-021-04970-5)
Supplement: Supplementary file 1 — Additional file 1: Table S1. PCR cycles for the ITS (ITS1-5.8S rRNA-ITS2) region. [file 13071_2021_4970_MOESM1_ESM.docx]

**Additional file 1: Table S1.** PCR cycles for the ITS (ITS1-5.8S rRNA-ITS2) region.

| Primer | Initial Denaturation | | Denaturation | Annealing | Extension | Final Elongation | Cycles |
| --- | --- | --- | --- | --- | --- | --- | --- |
| CS249-  FL | | 3 min 93 °C | 1 min 93 °C | 1 min 50 °C | 1 min 72 °C | 10 min 72 °C | 30 |
| CAS18sF1-  CAS5p8sB1d | | 4 min 94 °C | 20 sec 95 °C | 40 sec 67 °C | 20 sec 72 °C | 2 min 72 °C | 35 |
| CAS5p8sFc-  CAS28sB1d | | 4 min 94 °C | 20 sec 95 °C | 40 sec 55 °C | 20 sec 72 °C | 2 min 72 °C | 35 |
