# Supplementary material for: Population genetics and phylogeography of Tabanus bromius (Diptera: Tabanidae)
Source: Parasit Vectors. 2021 Sep 6;14:453. doi: 10.1186/s13071-021-04970-5 (PMC8420036; doi:10.1186/s13071-021-04970-5)
Supplement: Supplementary file 2 — Additional file 2: Table S2. COI haplotypes and their frequencies in populations. [file 13071_2021_4970_MOESM2_ESM.docx]

**Additional file 2: Table S2.** COI haplotypes and their frequencies in populations.
