# Supplementary figures and images for: Population genetics and phylogeography of Tabanus bromius (Diptera: Tabanidae)
Source: Parasit Vectors. 2021 Sep 6;14:453. doi: 10.1186/s13071-021-04970-5 (PMC8420036; doi:10.1186/s13071-021-04970-5)

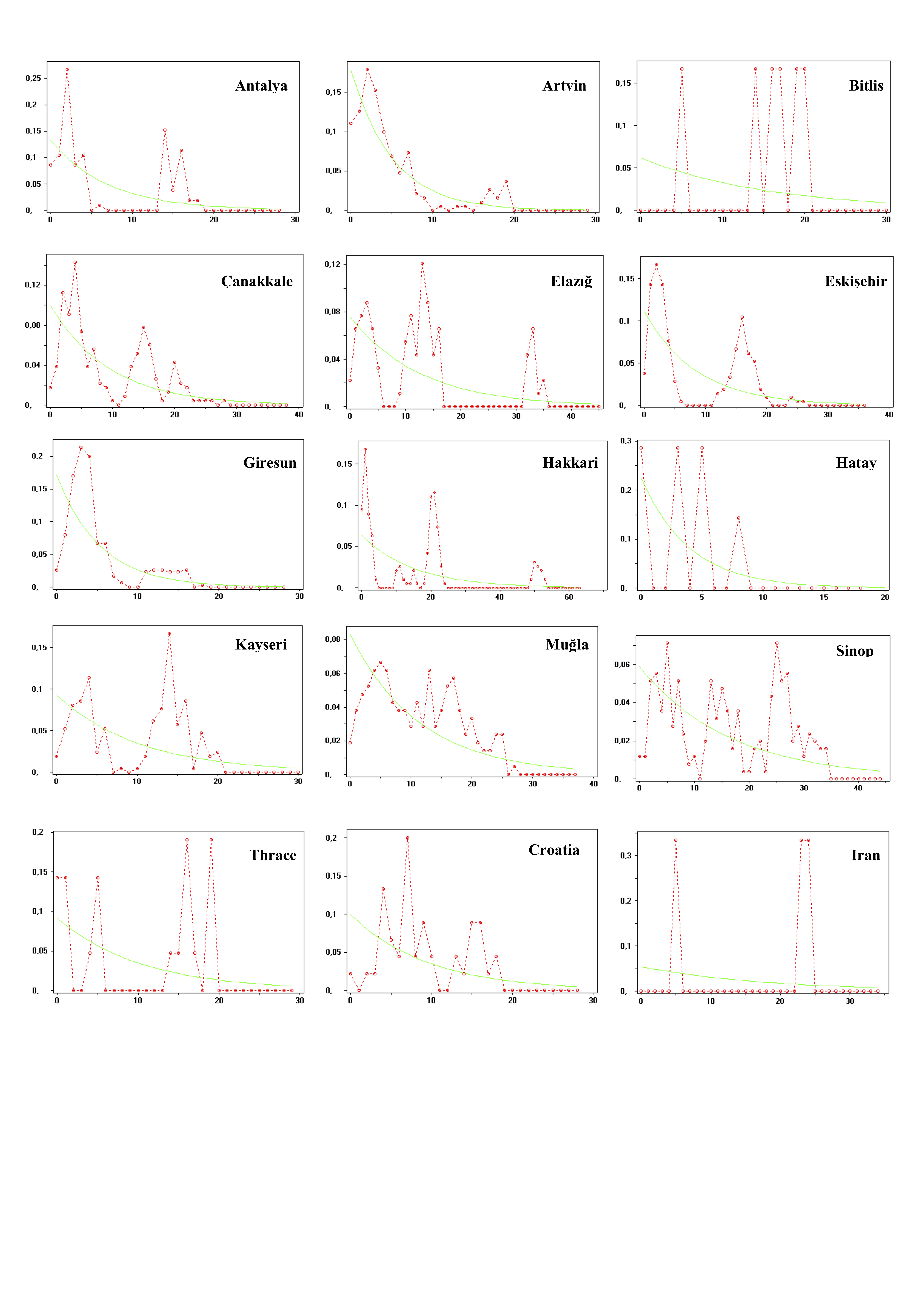

Supplement: Supplementary file 4 — Additional file 4: Figure S1. Mismatch distribution profile of each population obtained using the COI haplotypes. The observed distribution is represented by a red line (Obs), and the expected by a green line (Exp). [file 13071_2021_4970_MOESM4_ESM.tiff]

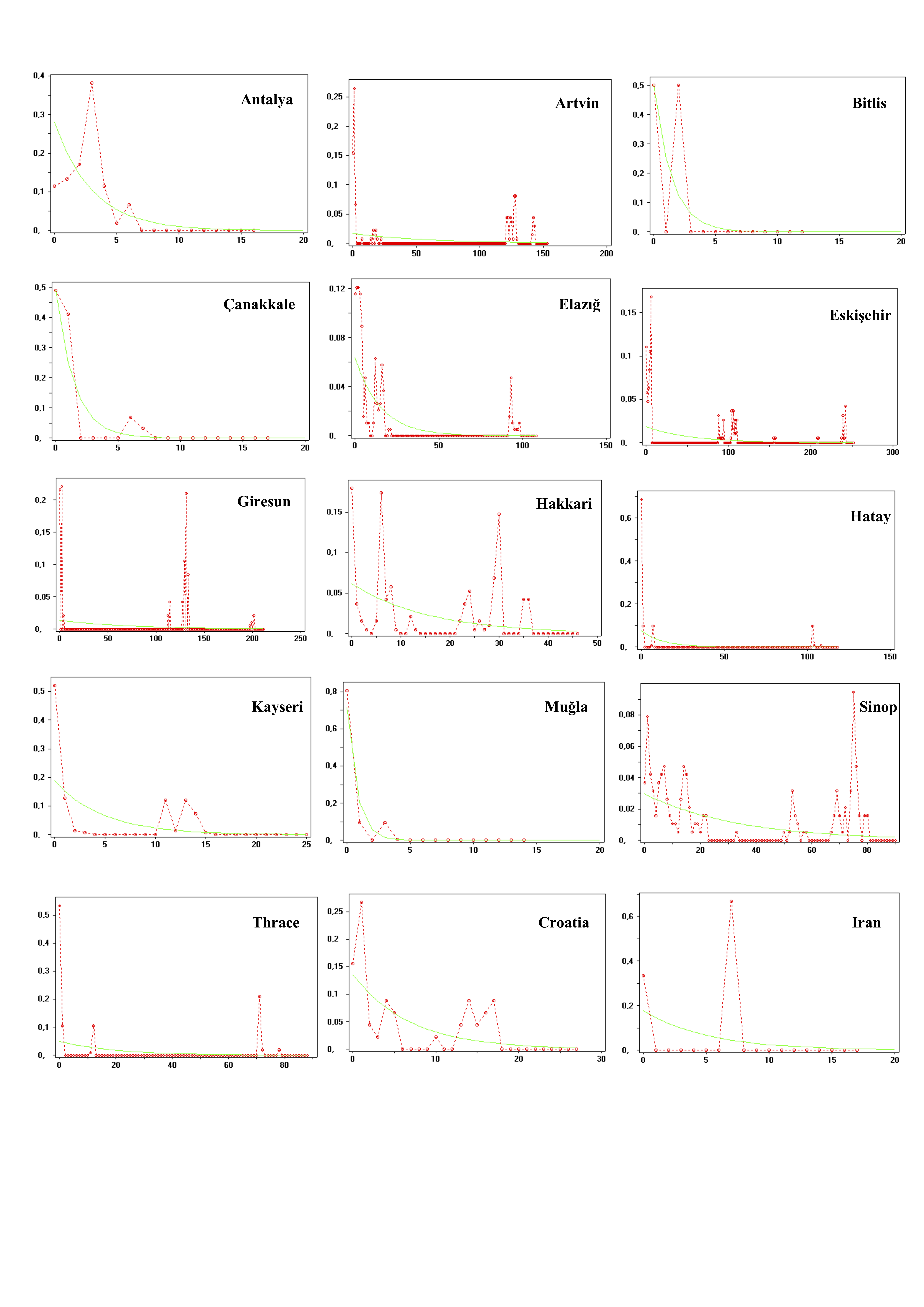

Supplement: Supplementary file 5 — Additional file 5: Figure S2. Mismatch distribution profile of each population obtained using the combined ITS1-ITS2 alleles. The observed distribution is represented by a red line (Obs), and the expected by a green line (Exp). [file 13071_2021_4970_MOESM5_ESM.tiff]
